# Metabolomics insights into doxorubicin and 5-fluorouracil combination therapy in triple-negative breast cancer: a xenograft mouse model study

**DOI:** 10.3389/fmolb.2024.1517289

**Published:** 2025-01-13

**Authors:** Mai M. Hassanein, Yousra A. Hagyousif, Ruba A. Zenati, Hamza M. Al-Hroub, Farman Matloob Khan, Ahmad Y. Abuhelwa, Karem H. Alzoubi, Nelson C. Soares, Waseem El-Huneidi, Eman Abu-Gharbieh, Hany Omar, Dana M. Zaher, Yasser Bustanji, Mohammad H. Semreen

**Affiliations:** ^1^ Department of Medicinal Chemistry, College of Pharmacy, University of Sharjah, Sharjah, United Arab Emirates; ^2^ Research Institute of Medical and Health Sciences, University of Sharjah, Sharjah, United Arab Emirates; ^3^ Department of Pharmacy Practice and Pharmacotherapeutics, College of Pharmacy, University of Sharjah, Sharjah, United Arab Emirates; ^4^ Laboratory of Proteomics, Department of Human Genetics, National Institute of Health Doutor Ricardo Jorge (INSA), Lisbon, Portugal; ^5^ Center for Applied and Translational Genomics (CATG), Mohammed Bin Rashid University Medicine and Health Sciences (MBRU), Dubai Health, Dubai, United Arab Emirates; ^6^ Department of Basic Medical Sciences, College of Medicine, University of Sharjah, Sharjah, United Arab Emirates; ^7^ Department of Clinical Sciences, College of Medicine, University of Sharjah, Sharjah, United Arab Emirates; ^8^ School of Pharmacy, The University of Jordan, Amman, Jordan

**Keywords:** triple-negative breast cancer, MDA-MB-231 xenograft model, untargeted metabolomics analysis, UHPLC-ESI-QTOF-MS, doxorubicin, 5-flurouracil

## Abstract

**Background:**

Breast cancer is one of the most prevalent malignancies and a leading cause of death among women worldwide. Among its subtypes, triple-negative breast cancer (TNBC) poses significant clinical challenges due to its aggressive behavior and limited treatment options. This study aimed to investigate the effects of doxorubicin (DOX) and 5-fluorouracil (5-FU) as monotherapies and in combination using an established MDA-MB-231 xenograft model in female BALB/C nude mice employing advanced metabolomics analysis to identify molecular alterations induced by these treatments.

**Methods:**

We conducted comprehensive plasma and tumor tissue sample profiling using ultra-high-performance liquid chromatography-electrospray ionization quadrupole time-of-flight mass spectrometry (UHPLC-ESI-QTOF-MS).

**Results:**

Each treatment group exhibited unique metabolic profiles in plasma and tumor analysis. Univariate and enrichment analyses identified alterations in metabolic pathways. The combination treatment of DOX + 5-FU induced the most extensive metabolic alterations disrupting key pathways including purine, pyrimidine, beta-alanine, and sphingolipid metabolism. It significantly reduced critical metabolites such as guanine, xanthine, inosine, L-fucose, and sphinganine, demonstrating enhanced cytotoxic effects compared to individual treatments. The DOX treatment uniquely increased ornithine levels, while 5-FU altered sphingolipid metabolism, promoting apoptosis.

**Significance:**

This *in vivo* study highlights TNBC’s metabolic alterations to chemotherapeutics, identifying potential biomarkers like L-fucose and beta-alanine, and provides insights for improving treatment strategies.

## 1 Introduction

Breast cancer (BCa) is the most prevalent cancer among women worldwide and a leading cause of death. In 2020 the WHO reported 2.3 million new cases, representing 11.7% of all cancers and 685,000 deaths. In the UAE, breast cancer accounted for 21.4% of cancer cases, with 1,030 new diagnoses (Ferlay et al., 2021). Among BCa subtypes, triple-negative breast cancer (TNBC) is particularly aggressive, making up 15%–20% of cases, and more common in younger women and those with BRCA1 variants (Siegel et al., 2023; Sharma, 2016). TNBC represents a complex and challenging subtype due to its aggressive nature, limited treatment options, and poorer prognosis in comparison with other BCa subtypes (Baranova et al., 2022; Won and Spruck, 2020; Chang et al., 2019). It lacks estrogen receptor (ER), progesterone receptor (PR), and human epidermal growth factor receptor 2 (HER2) expression, rendering hormonal and HER2-targeted therapies ineffective (Prat et al., 2013).

The Standard BCa treatment including surgery, chemotherapy, radiation, and other therapies, is tailored based on the patient’s condition and disease stage (Lee et al., 2011; Joensuu and Gligorov, 2012; Yin et al., 2020; Miller et al., 2022). Despite advancements in understanding BCa, developing effective targeted therapies for TNBC remains a significant challenge (Joensuu and Gligorov, 2012; Yin et al., 2020). Chemotherapy has emerged as the primary approach for TNBC treatment (Bianchini et al., 2016). The neoadjuvant chemotherapy regimens have shown a promising, significant high rate of pathological remission, leading to improved prognosis of TNBC (Sharma, 2016; Murphy et al., 2018; Holanek et al., 2021). Several combinations are recommended by The National Comprehensive Cancer Network (NCCN) guidelines for the management of TNBC, including taxane, anthracycline, cyclophosphamide, cisplatin, and fluorouracil (Sharma, 2016; Hsu et al., 2022). Doxorubicin (DOX) and 5-fluorouracil (5-FU) are commonly used chemotherapy agents in breast cancer treatment (Balmer et al., 2014). DOX, an anthracycline antibiotic, intercalates DNA and inhibits topoisomerase II enzyme, impeding DNA replication and inducing apoptosis in cancer cells (Yang et al., 2014; Tacar et al., 2012). 5-FU is a pyrimidine analog that inhibits thymidylate synthase, an enzyme critical for DNA synthesis, thereby disrupting cancer cell proliferation (Aggarwal et al., 2021). These chemotherapeutics have shown efficacy in various cancers, but their limited effectiveness as monotherapies in TNBC highlights the need for combination therapies to improve treatment response, as recommended by NCCN guidelines (Joensuu and Gligorov, 2012; Zoli et al., 2005; Martin et al., 2021).

Recurrence and therapeutic resistance pose significant obstacles in TNBC treatment. Targeting BCa-specific metabolic vulnerabilities would be a promising approach to overcome these obstacles (Takahashi et al., 2013). Metabolomics research facilitates the investigation of metabolic profiles, providing insights into the dynamic changes occurring in metabolic pathways in response to treatment which enhances our understanding of drug sensitivity or resistance mechanisms (Schmidt et al., 2021). Moreover, metabolomics analysis offers valuable information about the complex interrelationships between the tumor’s metabolism and its response to therapeutic interventions, enabling the identification of novel therapeutic targets and strategies for improved TNBC management (Shajahan-Haq et al., 2015; Ogrodzinski et al., 2017; Judes et al., 2016). Despite advances, innovative therapeutic approaches for TNBC management are urgently needed. In response, omics techniques such as genomics, transcriptomics, proteomics, and metabolomics have been developed to identify genes, mRNA, proteins, and metabolites (Manzoni et al., 2018). These methods have improved early disease detection and revealed indicators of underlying disease processes (Valenti et al., 2021).

Many studies have examined omics applications in the field of Bca research; however, limited research has been conducted to assess the influence of anticancer drugs on the metabolism of breast cancer cells (Semreen et al., 2020). In a previous study, we investigated the metabolic changes in MCF7 and MDA-MB-231 cancer cells after treatment with Tamoxifen and/or Paclitaxel (Semreen et al., 2020). In this study, we aim to investigate the effects of DOX and 5-FU, both as individual treatments and in combination, using the MDA-MB 231 cell line-derived xenograft (CDX) model of TNBC and to employ untargeted metabolomics analysis using UHPLC-ESI-QTOF-MS, to elucidate the metabolic alterations induced by these treatments. The comprehensive metabolomic profiling of plasma and tumor tissue samples and tumor growth assessment provides valuable insights into these treatments’ efficacy and potential enhanced effects in TNBC.

## 2 Materials and methods

### 2.1 Cell culture growth conditions

MDA-MB-231 cell line was cultured in DMEM medium (5.55 mM glucose and 1 mM sodium pyruvate), supplemented with 10% fetal bovine serum and 1% penicillin/streptomycin (Sigma Aldrich, St. Louis, MO, United States) at 37°C in a humidified atmosphere containing 5% CO_2_.

### 2.2 MDA-MB 231 xenograft mice model

This study was approved by the Institutional Animal Care and Use Committee (IACUC) at the University of Sharjah (Ethical Approval Number: ACUC-06-08-2022). Standard ethical guidelines were followed in all animal procedures conducted.

Thirty healthy female BALB/C nude mice (8–10 weeks age), with an average weight of 26–27 g, were included in the study and maintained in our institutional animal facility following the animal care guidelines. A detailed justification of the sample size used in this study, including the rationale based on the resource equation method, is provided in Supplementary Data S1 (Charan and Kantharia, 2013). Animal welfare was carefully monitored daily by assessing the cage environment, behavior, and physical appearance. Mice were housed in groups of three per cage and provided with easily accessible food and water. The mice were randomized into five experimental groups (6 per group). Twenty-four mice were selected to establish the (CDX) model using MDA-MB 231 cells, and the remaining six mice were designated as the negative control, consisting of healthy mice not injected with MDA-MB-231 cells and did not develop tumors. MDA-MB-231 cells (2 × 10^6^ in 50 μL PBS & 50 μL Matrigel) were injected subcutaneously into the neck region of the mice to induce tumor formation. Tumor-bearing mice reached a tumor volume of 150–200 mm^3^ approximately 6 weeks post-implantation, after which they were randomized into four groups: untreated xenografts consisting of mice with tumors that received no treatment referred to as positive control group) and three treatment groups, DOX, 5-FU, and a combination of DOX and 5-FU. The mice in the DOX group were administered 1 mg/kg of DOX once weekly (Sudha et al., 2017), and the mice in the 5-FU group received 50 mg/kg of 5-FU daily for five consecutive days and both treatments were administered via an intraperitoneal (i.p.) route (Lim et al., 2019). The mice in the combination therapy group received DOX & 5-FU as separate injections, and the treatment duration was 2 weeks. The body weight of the mice was measured at the start of the study and twice weekly from the beginning of the treatments. Tumor growth and progression were monitored by palpation and measurement of tumor size periodically using a digital vernier caliper. The tumor volume in cm^3^ was determined using the formula (volume = π/6 × length × width^2^) (Yuan et al., 2016). All mice were anesthetized and humanely euthanized at the end of the treatment period. Tumors were excised and weighed, and their sizes were measured. Also, blood plasma was collected from each mouse. Both tumor and plasma samples were stored at −80°C for further analysis. During sample collection, two mice were excluded due to the low plasma volume obtained, resulting in a final plasma analysis of 28 samples and 22 samples for the tumor analysis. The distribution of samples in the final analysis was as follows: DOX (n = 6), 5-FU (n = 5), DOX & 5-FU (n = 5), Positive Control (n = 6), and Negative Control (n = 6). All other data points and animals were included as planned. Additional details are provided in Supplementary Data S2.

### 2.3 Preparation of the samples for metabolomics extraction

#### 2.3.1 Metabolites extraction from plasma

Each collected plasma sample (100 µL) was mixed with 300 µL of methanol (≥99.9%, LC-MS CHROMASOLV) in Eppendorf tubes, followed by vortex and incubation at −20°C for 2 h. After vortex and centrifugation at 14,000 rpm for 15 min, the supernatant evaporated at 35°C–40°C using speed vacuum evaporation (EZ-2 Plus (GeneVac, Ipswich, United Kingdom). The extracted samples were resuspended in 200 µL of 0.1% formic acid in Deionized Water-LC-MS CHROMASOLV. Subsequently, the supernatant was filtered through a 0.45 µm pore size hydrophilic nylon syringe filters and collected in inserts within LC glass vials for LC-MS/MS analysis. All samples were placed in the autosampler at the temperature set at 4°C to proceed with the analysis by Q-TOF MS. A pooled QC sample was created to ensure analysis reproducibility by mixing 10 µL from each sample.

#### 2.3.2 Metabolites extraction from tumor

Tumor tissue was mechanically homogenized to prepare it for analysis. Preparation involved cutting frozen tumors into pieces and mixing 75 mg of each tumor tissue with 300 µL of lysis buffer and protease inhibitor. Ultrasonic homogenization was performed multiple times for cell disruption and to release metabolites and cellular components essential for metabolic analysis using the COPLEY sonicator or QSONICA SONICATOR (Qsonica, Newtown, CT, United States) under 90% amplifier and for 30 s with an ice bath employed throughout the process, after centrifugation (15,000 rpm, 10 min, 4°C), the supernatants were collected and further processed following the same plasma sample preparation protocol mentioned above.

### 2.4 UHPLC-ESI-QTOF-MS metabolic plasma profiling and tumor profiling

Metabolic profiling of plasma and tumor samples was performed using a UHPLC-ESI-QTOF-MS system, which enabled the identification of distinct metabolic alterations across different treatment groups. The analysis followed a data-dependent acquisition method in positive ion mode, ensuring high precision and reliable identification of metabolites. Quality control (QC) injections were conducted at regular intervals to ensure consistency throughout the experiment. Technical specifications and methodology for UHPLC-ESI-QTOF-MS are detailed in Supplementary Data S3.

### 2.5 Data processing and statistical analysis

Metabolite datasets obtained from the UHPLC-ESI-QTOF-MS analysis were processed using MetaboScape® 4.0 software (Bruker Daltonics, Billerica, MA, United States). All Metabolites included were matched against the Human Metabolome Database (HMDB). MetaboAnalyst® 5.0 software was used for data processing, including normalization, transformation, and quality checks like missing value imputation and outlier detection. It was also used to perform multivariate analysis using sparse Partial Least Squares - Discriminant Analysis (sPLS-DA), and volcano plots to compare the study groups’ results. Univariate analysis: One-way ANOVA followed by Fisher’s *post hoc* test was conducted to compare the groups, an independent Student’s t-test, and a fold change analysis between each pair of groups. The fold change was adjusted at 1.5, and metabolites with a p-value <0.05 were considered statistically significant. Enrichment analysis was carried out for statistically significant metabolites. The statistical analysis of tumor assessment was conducted using GraphPad Prism 10 and the dplyr package. Tumor growth among different experimental groups was conducted using two-way ANOVA followed by Dunnett multiple comparisons test at adjusted *p*-value <0.05 (95% confidence interval). All data including the raw QGD files, has been deposited to Metabolomics Workbench (https://www.metabolomicsworkbench.org). The data track id is 4,449 and 4,450. Schematic representation of the design and workflow of the experimental xenograft model see Supplementary Figure S1.

## 3 Results

### 3.1 Tumor assessment

**FIGURE 1:**
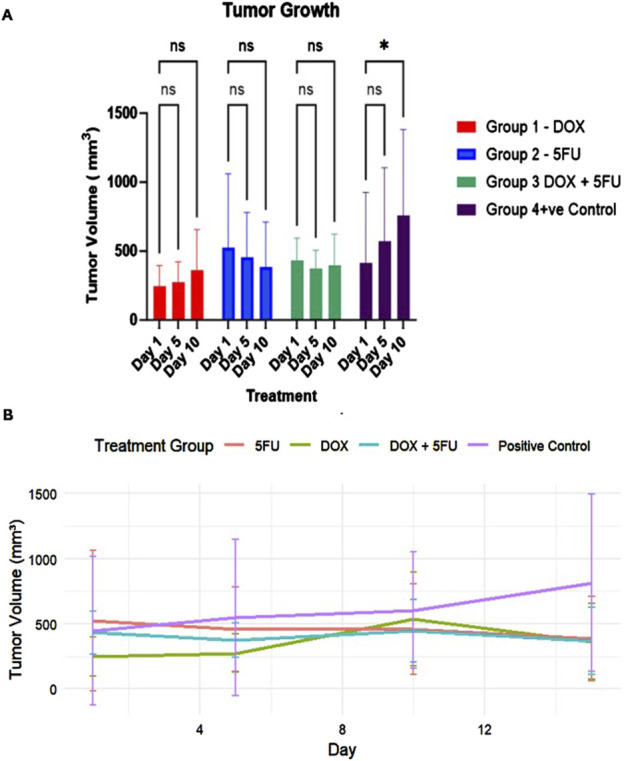
Tumor growth dynamics across different treatment groups over the 14-day study period. **(A)** Tumor volume measurements (mean ± standard deviation) at Days 1, 5, and 10 for Group 1 (DOX, red), Group 2 (5FU, blue), Group 3 (DOX + 5FU, green), and Group 4 (Positive Control, purple). Statistical significance is indicated by * (*p-value* < 0.05), while “ns” denotes no significant difference. **(B)** Tumor growth curve depicting the changes in tumor volume (mean ± standard deviation) for all groups throughout the treatment period. The positive control group exhibits continuous tumor growth, while treatment groups show restricted growth, with the combination therapy demonstrating the most pronounced effect.

The tumor assessment across the four distinct groups revealed the effectiveness of treatments used in restricting tumor growth (Figures 1A, B). All the treated groups showed a delayed increase in tumor volumes compared to the significant growth in the positive control group. Effect size analysis indicated that 5FU had the most substantial impact, followed by the combination treatment, while DOX showed a moderate effect in reducing tumor growth (Supplementary Table S1).

### 3.2 Plasma and tumor tissue metabolic profiling

This study aimed to explore the metabolic changes induced by DOX, 5-FU, and DOX/5-FU combination treatments in MDA-MB-231 xenograft model using UHPLC-ESI-QTOF-MS to perform metabolic plasma and tumor profiling. Ninety-two metabolite datasets were annotated from plasma, and 202 metabolites were detected from the tumor tissues of the metabolomic analysis of plasmaand tumor tissues (Supplementary Figure S1). All Metabolites included were matched against the Human Metabolome Database (HMDB); accordingly, a total of 89 metabolites were included in the plasma analysis, and 190 metabolites were included in the tumor analysis. Venn diagram (Supplementary Figure S1) demonstrated 20 shared significant metabolites between plasma and tumor samples, indicating overlapping metabolic alterations, and highlighting distinct changes unique to each sample type. Each treatment showed unique distinct metabolic profiles.

**FIGURE 2:**
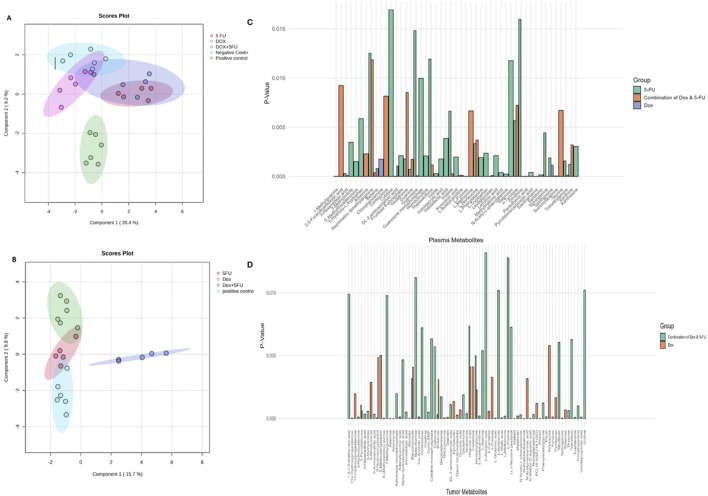
Multivariate analysis and metabolite comparisons across treatment groups. **(A)** sPLS-DA scores plot for plasma metabolomics data showing separation of treatment groups along Component 1 (20.4%) and Component 2 (9.2%). Distinct clustering of the DOX, 5FU, and DOX + 5FU groups is observed, with partial overlaps suggesting shared metabolic responses. **(B)** sPLS-DA scores plot for tumor metabolomics data showing group separation along Component 1 (15.7%) and Component 2 (9.8%). The DOX + 5FU group forms a distinct cluster, reflecting unique metabolic alterations induced by the combination treatment. **(C)** Histogram of significantly altered plasma metabolites for each treatment group (DOX, 5FU, and DOX + 5FU), with p-values indicating statistical significance. **(D)** Histogram of significantly altered tumor metabolites for DOX and DOX + 5FU treatments, highlighting key metabolic changes unique to each treatment.

A multivariate analysis using sPLS-DA, as shown in Figure 2, highlights various metabolic clustering among the treatment groups, emphasizing the specific effects of individual treatments, the significance of 5-FU in the combination therapy, and potential shared metabolic responses in the positive control and combined groups. In the metabolomics analysis of plasma (Figure 2A), the sPLS-DA results show that the DOX-treated group demonstrates a distinct separation from all other groups. Notably, the 5-FU group fully overlaps with the combined treatment group, indicating a potential dominant influence of 5-FU in the combination therapy. The positive control and combined treatment groups exhibit metabolic characteristics suggesting a more comprehensive response to tumor growth or xenograft conditions, independent of the applied treatments. Additionally, partial overlap between the positive and negative controls suggests inherent metabolic similarities. The classification error rate for this analysis, evaluated using 5-fold cross-validation, decreased progressively with an increasing number of components, starting at 64.3% for one component and improving to 32.1% with five components (Supplementary Figure S2A). This indicates the model achieves optimal separation with five components, demonstrating its robustness in classifying treatment groups.

In the metabolomic analysis of tumors (Figure 2B), the sPLS-DA model revealed that the combined treatment group forms a distinct cluster, reflecting unique metabolic alterations induced by the DOX and 5-FU combination. The positive control fully overlaps with the 5-FU group cluster, suggesting similarities in metabolic responses. DOX and 5-FU groups show limited intersections, indicating partly distinct metabolic profiles. The classification error rate for this analysis also followed a clear trend: it initially remained at 36.4% for the first two components, improved significantly to 18.2% with three components, and then increased to 22.7% and 31.8% with four and five components, respectively (Supplementary Figure S2B). This pattern suggests that the model achieves its best performance with three components, effectively balancing classification accuracy and model complexity. In addition to the multivariate clustering observed in plasma and tumor metabolomics data, detailed comparisons of significantly altered metabolites are shown in Figures 2C, D.

Univariate analysis using Student’s t-test was used to detect significantly altered metabolites by comparing the effect of each drug treatment on every single metabolite to that of the positive control group, followed by an enrichment analysis for the significantly altered metabolites identified. Alterations in the metabolic profiling of the plasma and tumor compared to the positive control group are summarized in Supplementary Figures S2, S3.

In the plasma metabolomic analysis, 89 metabolites were included after data curation and filtration (see M&M Section 2.5). Among them, DOX treatment showed only one significantly elevated, ornithine metabolite, and three reduced metabolites (Supplementary Figure S3A). In contrast, the 5-FU treatment exhibited two elevated metabolites indolelactic acid and sphinganine, and 20 reduced metabolites including guanine, guanosine, xanthine, and xanthosine (Supplementary Figure S3B). After DOX + 5-FU combination treatment, only one metabolite, cortexolone, was elevated, and the other had 16 reduced metabolites (Supplementary Figure S3C). Enrichment analysis revealed affected metabolic pathways, including glycine and serine metabolism, spermidine and spermine biosynthesis for DOX, and purine, tryptophan, spermidine, and spermine biosynthesis for 5-FU.

**FIGURE 3:**
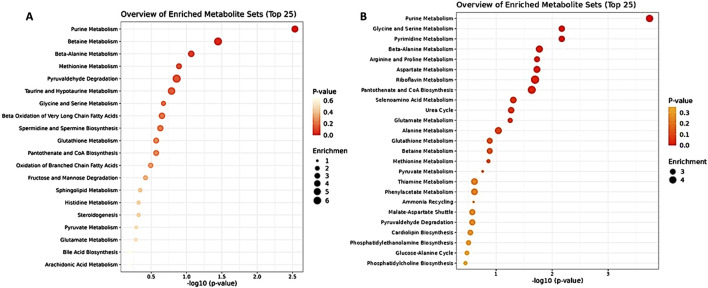
Enrichment Analysis of DOX + 5 FU and positive control of plasma metabolites (left) and tumor metabolites (right).

The metabolic tumor profiling analysis included 190 metabolites after data curation and filtration (see M&M Section 2.5), and in the DOX-treated group, 15 elevated and five reduced metabolites were identified (Supplementary Figure S3D). For the DOX/5-FU combination, 20 elevated and 28 reduced metabolites were detected, as shown in Supplementary Figure S3E. The most affected pathways were amino sugar, purine, thiamine, methionine, histidine metabolism, and malate-aspartate shuttle for DOX, and purine, glycine, serine, pyrimidine, beta-alanine metabolism, riboflavin, aspartate, arginine, and proline metabolism for the DOX/5-FU treated group. Notably, 5-FU treatment showed no significantly altered metabolites in the tumor analysis. The enrichment analysis comparing the plasma and tumor analyses in response to DOX + 5-FU treatment revealed significant impacts on similar metabolic pathways, including purine and glycine metabolism (Figure 3). The most pronounced metabolic pathway alterations and key metabolite changes identified across the different treatment groups (DOX, 5-FU, and their combination) in plasma and tumor tissues are summarized in Table 1, highlighting their significance in TNBC progression and potential as biomarkers for therapeutic efficacy.

**TABLE 1:** Summary of metabolic pathway alterations and key metabolite changes in plasma and tumor tissues across different treatment groups in the TNBC xenograft model.

Treatment group	Top enriched metabolic pathway	Altered metabolites	Significance on TNBC	Potential as biomarkers
DOX	Polyamine Biosynthesis, Amino Sugar, Methionine, and Histidine Metabolism	Plasma: Ornithine (↑), Sphingosine (↓) Tumor: Sphinganine (↑), Putrescine (↑)	Ornithine elevation suggests disruption in polyamine biosynthesis and potential ODC inhibition, enhancing DOX’s tumor-killing effects	Ornithine, Methionine, and Histidine (Indicators of DOX treatment response and metabolic vulnerabilities)
			Alterations in methionine and histidine metabolism imply changes in amino acid metabolism, affecting cellular energy and antioxidant defenses	
5-FU	Pyrimidine and Purine Metabolism, Sphingolipid Metabolism	Plasma: Guanine (↓), Xanthine (↓), Inosine (↓), Sphingosine (↓) Tumor: Sphinganine (↑)	Altered pyrimidine and purine metabolism indicate 5-FU’s impact on nucleotide synthesis pathways, affecting TNBC cell proliferation	Sphinganine, Sphingosine, and Purine metabolites (Potential biomarkers for 5-FU efficacy and tumor adaptation)
			Changes in sphingolipid metabolism, particularly sphinganine elevation in tumors, suggest involvement in apoptosis regulation	
Combined (DOX + 5-FU)	Purine, Pyrimidine, Beta-Alanine, Sphingolipid, Glycine, Serine, and Urea Cycle Metabolism	Plasma: L-Fucose (↓), Anserine (↓), Sphinganine (↓) Tumor: Guanine (↓), Xanthine (↓), Inosine (↓), Adenosine (↑), Sphingosine (↓), Sphinganine (↓), Ureidopropionic acid (↓)	The combination treatment disrupts multiple pathways, affecting DNA/RNA synthesis, oxidative stress balance, cellular repair, energy production, and apoptosis regulation, suggesting broad metabolic interference in TNBC growth and survival	L-fucose, Sphingosine, Anserine, (Potential biomarkers for combination treatment efficacy and metabolic vulnerabilities in TNBC)

Footnote: This table provides a comprehensive summary of the most significant metabolic pathway alterations observed in plasma and tumor tissues across different treatment groups (DOX, 5-FU, and their combination) in the TNBC, xenograft model. The arrows (↑/↓) indicate whether the metabolites were elevated or reduced following treatment. The identified metabolites’ potential roles as biomarkers highlight their significance in understanding TNBC’s metabolic vulnerabilities and response to therapy.

## 4 Discussion

Triple-negative breast cancer (TNBC) is a highly aggressive, challenging, and hard-to-treat type of breast cancer. Its inherent biological heterogeneity, along with the marked high potential of metastasis and poor prognosis, renders current treatments less effective and further complicates the identification of effective therapeutic regimens (Yuan et al., 2016). Despite progress in understanding TNBC metabolism, important gaps persist. Cancer research has revealed that various metabolic pathways involved in energy production and biosynthetic needs are crucial for cancer cells’ rapid growth and proliferation. Several amino acids have been identified to have characteristically high levels in different types of cancer compared to normal tissue (Zenati et al., 2023). However, much of the current research depends on cancer cell lines, which do not fully replicate the complex tumor microenvironment, leading to variability and limiting clinical relevance. Addressing these limitations requires integrating studies utilizing both model systems and human patients. Research on TNBC’s metabolic response to different chemotherapeutics is still limited, particularly concerning combination therapies. Understanding how TNBC adapts metabolically to these treatments is crucial, especially given the associated side effects and resistance. Using animal models is vital for examining these interactions in a more clinically relevant context, aiming to develop more effective and targeted treatment strategies.

Our study used untargeted metabolomics to evaluate the alterations in metabolites and metabolic pathways of the MDA-MB-231 breast cancer xenograft model following treatment with DOX and/or 5-FU (Table 1). We utilized the MDA-MB-231 cell line, classified as an MSL Basal B TNBC subtype, which is widely used in xenograft models to investigate tumor growth, metastasis, and therapeutic responses (Ahmed, 2009; Junior et al., 2022). Based on our hypothesis, we performed both tumor tissue and plasma profiling to comprehensively understand metabolic alterations in TNBC. While plasma profiling provides insights into systemic metabolic changes, it can be affected by factors such as diet, lifestyle, and medication, making it less accurate in reflecting tumor-specific metabolism. Studies have shown that metabolite levels in biofluids do not always correlate with those in tumor tissues (Lehmann et al., 2011). To overcome this, we included tumor tissue profiling to obtain more precise tumor-specific metabolic information. Our findings confirmed this approach, as a greater number of metabolites were identified in tumor tissues after treatment, revealing distinct metabolic profiles across different treatment groups (Supplementary Figure S1).

The multivariate analysis revealed that DOX, 5-FU, and their combination induced distinct metabolic alterations, with each treatment showing unique profiles. Plasma analysis indicated elevated ornithine levels with DOX treatment, while the combination therapy mirrored the metabolic changes seen with 5-FU, suggesting a dominant influence of 5-FU in the combination. Tumor tissue analysis supported this, showing a distinct metabolic signature for the combination treatment (Supplementary Figure S3E), with no significant alterations observed in the 5-FU group, indicating a different response within the tumor microenvironment.

The sPLDA analysis further highlighted these findings, showing a complete overlap between the 5-FU and combination treatment groups in plasma, suggesting 5-FU’s effects overshadowed DOX when combined. The partial overlap between control and treatment groups in both plasma and tumor analyses suggests shared metabolic characteristics related to tumor growth or the inherent metabolic state of the xenograft models (Figures 2A, B).

Our study identified key metabolic pathways altered in TNBC treatment, with purine metabolism, spermidine, and spermine biosynthesis among the top enriched pathways across different treatment groups in plasma analysis. Tumor tissue profiling revealed more complex metabolic changes, especially in the combination DOX + 5-FU group, where purine, pyrimidine, and amino acid pathways, including glycine, serine, and beta-alanine metabolism, were significantly enriched (Supplementary Figure S3F). In contrast, the DOX treatment group showed distinct alterations in purine, amino sugar, methionine, and histidine metabolism, pathways crucial for cellular energy production, nucleotide synthesis, and antioxidant defense. These findings align with known metabolic alterations in TNBC, such as increased glycolysis (Warburg effect), enhanced serine biosynthesis, dependence on glutaminolysis, and altered lipid metabolism, which collectively contribute to the aggressive behavior and growth of TNBC (Kazan et al., 2019; Ireson et al., 2019; Mattaini et al., 2016; Antalis et al., 2010).

In the group treated with the combination of DOX + 5FU, we observed substantial reductions in key purine metabolites such as guanine, xanthine, and inosine, along with increased levels of adenosine and its derivatives, suggesting disruptions in purine synthesis critical for DNA and RNA production in rapidly proliferating cancer cells.

These findings align with 5-FU’s known mechanism of action, as the drug primarily targets pyrimidine metabolism but also impacts purine metabolism through its effects on one-carbon (1C) metabolism and the folate cycle. By inhibiting thymidylate synthase (TYMS) via its active metabolite FdUMP, 5-FU interferes with the conversion of dUMP to dTMP, impairing DNA synthesis and indirectly affecting purine synthesis by reducing the availability of tetrahydrofolate (THF) derivatives, crucial for *de novo* purine production. This dual inhibition of both pyrimidine and purine pathways significantly disrupts nucleotide synthesis, contributing to the cytotoxic effects observed in TNBC cells treated with 5-FU (Possemato et al., 2011; Choi et al., 2013).

The metabolic profiling also highlighted significant alterations in sphingolipid metabolism, particularly in sphingosine and sphinganine levels, with notable changes observed in the combination treatment group. Sphinganine levels were markedly reduced in the combination group but elevated in the 5-FU treatment, while sphingosine levels decreased in both DOX and 5-FU treated groups. Additionally, sphinganine was significantly elevated in tumor tissues of the DOX-treated group, indicating a unique metabolic response. These findings suggest that modulation of sphingolipid metabolism may contribute to the pro-apoptotic effects of DOX and 5-FU treatments. This is supported by previous studies demonstrating that sphinganine and sphingosine play crucial roles in inducing apoptosis and inhibiting cell proliferation, particularly in breast and colon cancers (Stine et al., 2022). The ability of these metabolites to arrest the cell cycle at the G2/M phase and promote programmed cell death highlights their potential as chemopreventive and chemotherapeutic agents, emphasizing their potential as therapeutic targets or biomarkers (Ogrodzinski et al., 2017; Hii et al., 2021).

Notably, the plasma metabolic profiling of the combined DOX and 5-FU treatment, revealed reduced L-fucose levels in plasma, suggesting a disruption in fucosylation processes that influence tumor behavior. This aligns with the critical role of L-fucose in cancer biology, where altered fucosylation is linked to metastasis and immune evasion in breast cancer (Bae et al., 2011; Markowski et al., 2023). These changes highlight L-fucose’s potential as a biomarker for treatment efficacy and a target for therapeutic intervention in TNBC.

The comparative analysis of plasma and tumor tissues also revealed overlapping effects on purine, pyrimidine, and beta-alanine metabolism, with beta-alanine metabolism emerging as one of the most enriched pathways in the combined treatment group (Supplementary Figure S3F). This is consistent with existing research showing that beta-alanine metabolism is more pronounced in ER-negative breast cancer and is linked to aggressiveness and poorer outcomes (Cheng et al., 2015). We observed alterations in related metabolites, such as anserine, pantothenic acid (PA), and ureidopropionic acid, which play key roles in oxidative stress response, energy production, and membrane repair. The reduction in plasma anserine levels may indicate a shift in oxidative balance, as anserine has antioxidant properties that protect against oxidative stress, a critical factor in tumor progression (Keeley et al., 2019). Ureidopropionic acid, a precursor to beta-alanine, was altered, suggesting changes in beta-alanine availability. Beta-alanine itself is a precursor for PA, essential for coenzyme A (CoA) synthesis, which plays a key role in the TCA cycle, fatty acid biosynthesis, and membrane phospholipid synthesis. Alterations in PA levels can influence energy production, cellular repair, and membrane integrity, which are vital for cancer cell survival (Budczies et al., 2013; Ihara et al., 2019).

These findings underscore the impact of the combined treatment on disrupting essential metabolic pathways in TNBC, offering insights into potential biomarkers and therapeutic targets for this aggressive subtype.

In the DOX-treated group, we observed elevated levels of ornithine in plasma, indicating a significant alteration in polyamine biosynthesis (Supplementary Figure S3A). The increase in ornithine suggests a potential inhibition of ornithine decarboxylase (ODC) activity, the enzyme responsible for converting ornithine to putrescine, a precursor for polyamine synthesis (Geck et al., 2020). Targeting ODC may reduce polyamine levels, which are essential for cancer cell growth, potentially enhancing the tumor-killing effects of DOX. Previous studies support this finding, showing that genotoxic chemotherapy, such as DOX or cisplatin, upregulates ornithine while reducing putrescine and spermidine levels, disrupting the cell cycle and making cancer cells more susceptible to DNA damage (Ma et al., 2020; Hutschenreuther et al., 2013). This disruption may increase DOX’s therapeutic efficacy, highlighting its potential as a strategy to target TNBC’s metabolic vulnerabilities.

Overall, our findings provide a foundation for identifying metabolic vulnerabilities, exploring the effects of combined chemotherapy treatments, and highlighting the importance of considering the tumor context and its metabolic adaptations to therapeutic interventions specific to TNBC. We plan to expand our research to include additional TNBC models to address heterogeneity and integrate proteomic data to explore correlations with metabolic alterations. This approach aims to uncover pathways and mechanisms triggered by the treatments used. By bridging these findings, we strive to develop more effective and personalized therapeutic strategies for TNBC, ultimately translating our research into meaningful clinical benefits.

## Data Availability

The data presented in the study are deposited in the Metabolomics Workbench repository, project ID PR001852, available at https://www.metabolomicsworkbench.org/data/DRCCMetadata.php?Mode=Project&ProjectID=PR001852.
