# Supplementary material for: Metabolomics insights into doxorubicin and 5-fluorouracil combination therapy in triple-negative breast cancer: a xenograft mouse model study
Source: Front Mol Biosci. 2025 Jan 13;11:1517289. doi: 10.3389/fmolb.2024.1517289 (PMC11769812; doi:10.3389/fmolb.2024.1517289)
Supplement: Supplementary file 1 [file DataSheet1.docx]

Supplementary Material

# Supplementary (S1): Sample Size Justification

In this study, we used 30 female BALB/C nude mice, randomly divided into five experimental groups with 6 mice per group. The sample size was determined using the resource equation method to ensure the study was adequately powered while maintaining the ethical use of animals. This resulted in an error degrees of freedom (E) of E=30−5=25, slightly above the recommended range of 10 to 20, indicating sufficient replication to detect treatment effects.

Given the complexity and sensitivity of metabolomics profiling, where high-quality data is crucial, extra mice were included to account for any potential data loss or sampling difficulties. Variations in biological responses and the need for precise metabolite measurements necessitate additional samples to ensure robustness and reliability in the metabolic profiling data. Including these extra mice allows for adequate coverage in case of technical issues or unforeseen biological variability.

With five groups in total, the selected sample size allows for sufficient replication to detect treatment effects while minimizing the use of animals. This method was used as an alternative to power analysis given the exploratory nature of untargeted metabolomics in this study. This method aligns with established guidelines for animal study design, balancing scientific rigor and ethical considerations. The selected sample size was carefully considered to ensure that it is sufficient to detect significant metabolic alterations induced by the treatments, providing a strong foundation for future targeted studies.

**Formula**:

**E=N-G**

Where: N = Total number of animals G = Total number of groups

**Given Values**:

- Total number of animals (N) = 30
- Total number of groups (G) = 5 (DOX, 5-FU, Combination, Positive Control and Negative Control)

**Calculate E**:

E=30-5=25

**References:**

Charan, J., & Kantharia, N.D., How to calculate sample size in animal studies, Journal of Pharmacology & Pharmacotherapeutics. 4 (2013) 303-306. doi:10.4103/0976-500X.119726.

# Supplementary (S2): Supplemental Experimental Procedures on Xenograft Model Establishment

- **Minimization of Confounders**

Mice were housed in groups of three per cage, with each animal coded, and cages were labeled with these codes and treatment details. This ensured unbiased handling and consistent measurements across treatment groups. Treatments were administered at the same time each day to ensure consistency. Additionally, two researchers were involved in the process of measurement and treatment administration. One researcher administered the treatments while another monitored and recorded the measurements. This division of roles ensured accuracy in treatment administration and objective monitoring, enhancing the reliability of the data collected.

- **Animal Care, Monitoring, and Humane Endpoints**

In this study, several interventions and monitoring protocols were implemented to minimize pain, suffering, and distress in the animals. To control tumor growth and enhance animal welfare, Matrigel was used to facilitate tumor establishment and ensure consistent tumor volumes. To mitigate the potential adverse effects of chemotherapy, we selected the minimum effective dose of DOX (1 mg/kg) to limit toxicity while ensuring therapeutic efficacy. Mice were closely monitored for signs of adverse effects, including weight loss and diarrhoea, which were anticipated post-treatment, Weight loss was observed particularly in the group receiving the combination therapy of DOX and 5-FU. The animals' body weights were measured twice weekly throughout the 2-week treatment duration to track any significant changes.

Humane endpoints were established based on the monitoring of specific clinical signs, including rapid weight loss (>20% body weight), severe diarrhea, and significant behavioural changes. The mice were monitored daily for these signs, and any animal reaching these predefined humane endpoints would be humanely euthanized to prevent unnecessary suffering.

Due to the careful monitoring and anticipated adverse effects, all mice survived to the end of the treatment period.

# Supplementary (S3)

**Reagents**

DOX and 5-FU were purchased from (Sigma-Aldrich,Germany). MDA-MB-231 cell line was purchased from the European Collection of Authenticated Cell Cultures (ECACC). Formic acid was obtained from Fisher Chemical (Geel, Belgium, UK). Methanol (≥99.9 %), acetonitrile, and deionized water were LC-MS CHROMASOLV purchased from Honeywell (Wunstorfer Strasse, Seelze, Germany).

**Technical Specifications and Methodology for UHPLC-ESI-QTOF-MS Metabolic Profiling.**

The LC-MS/MS analysis was conducted using an Elute UHPLC system (Bruker Daltonik GmbH, Bremen, Germany) coupled with a QTOF mass spectrometer with an ESI source. The setup also included a solvent delivery system pump (Elute UHPLC HPG 1300), an autosampler, and a temperature-controlled column compartment. The computer operating system employed was Windows 10 Enterprise 2016 LTSB. Data management was facilitated using Bruker Compass HyStar 5.0 SR1 Patch1 (5.0.37.1), Compass 4.1 for otofSeries, and otofControl Version 6.2. Mobile phases A (water with 0.1% formic acid) and B (acetonitrile with 0.1% formic acid) were used with the following gradient elusion mode: 0 to 2 min, 1% B; 2 to 17 min, 1–99% B; 17 to 20 min, 99% B; 20 to 20.1 min, 99–1% B; 20.1 to 30 min, 1% B. The flow rate was 0.25 mL/min from 0 to 20 min, 0.35 mL/min from 20 min to 28.3 min, and 0.25 mL/min from 28.3 to 30 min. The sample injection volume was 10 μl, and separation occurred on a Hamilton® Intensity Solo 2 C18 column (2.1 × 100 mm, 1.8 µm) (Bruker Daltonik) at 35°C. The ESI source conditions were set with a capillary voltage of 4500 V, drying gas flow rate of 10.0 l/min at 220°C, nebulizer pressure of 2.2 bar, and the End Plate offset at 500 V. Sodium formate (10 mM) was injected at the beginning of each sample run and used as a calibrant for internal calibration during data processing. The MS acquisition process consisted of two phases. First, an auto MS scan lasting from 0 to 0.3 minutes was utilized for calibrating sodium formate. The second phase encompassed auto MS/MS scanning with CID acquisition, including fragmentation, which extended from 0.3 to 30 minutes. Both acquisition phases were conducted in positive mode at a rate of 12 Hz. The automatic mass scan range within each run spanned from 50 to 1300 m/z, with a precursor ion width of ±0.5, a cycle time of 0.5 seconds, and a threshold of 400 counts. Active exclusion was initiated after three spectra and lifted after 0.2 minutes. For MS2 acquisition, a data-dependent acquisition (DDA) approach was employed, with collision energy settings varying between 100% and 250% and being set at 20 eV.

TRX-2101/RT-28-calibrants from Nova Medical Testing Inc. for the Bruker T-ReX LC-QTOF were injected before sample analysis to assess the column's performance, reversed-phase liquid chromatography (RPLC) separation, multipoint retention time calibration, and the mass spectrometer. Additionally, TRX-3112-R/MS Certified Human serum solution for Bruker T-ReX LC-QTOF (provided by Nova Medical Testing Inc.) was prepared from pooled human blood and administered before sample analysis to validate the performance of the LC-MS instruments. The analysis followed a randomized sequence order, commencing with five injections of solvent A (0.1% formic acid in deionized water) to facilitate apparatus equilibration. Subsequently, five injections of the pooled QC sample were carried out. Furthermore, one QC injection was conducted every (9-10 samples) to assess the consistency of the analysis.

# Supplementary Figures and Tables

##
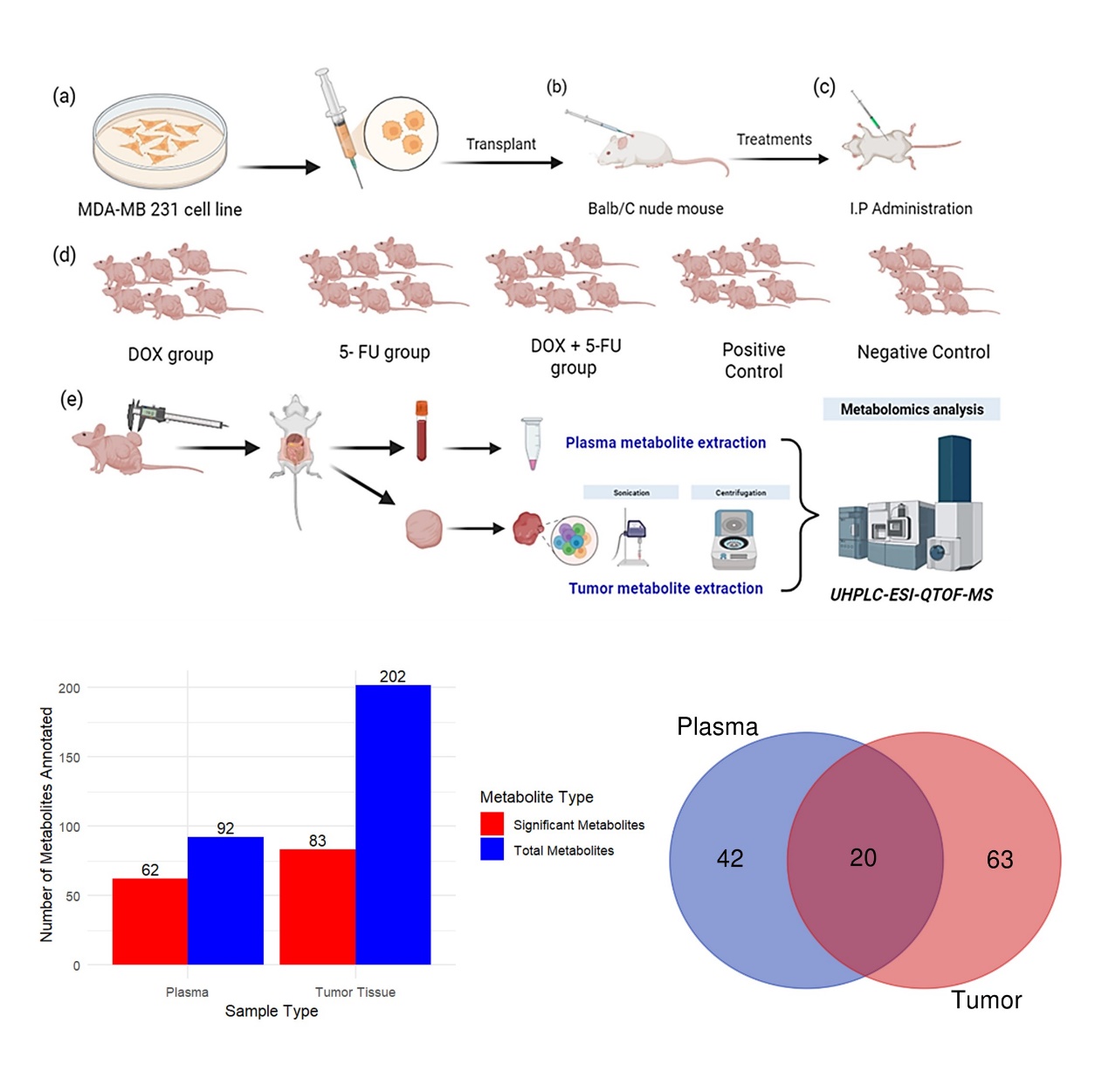
Supplementary Figures

Figure S1. Schematic representation of design and workflow of the Experimental xenograft. Total annotated metabolites and significant metabolites identified in plasma and tumor tissues following filtration and ANOVA post-hoc analysis with p-value < 0.05.


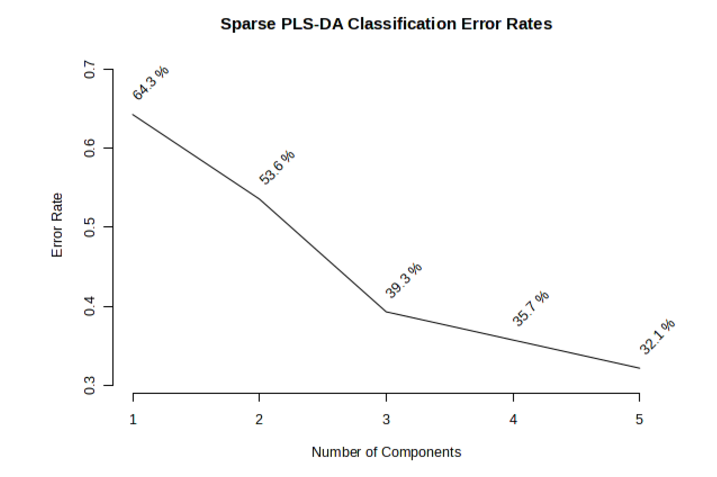


**A**


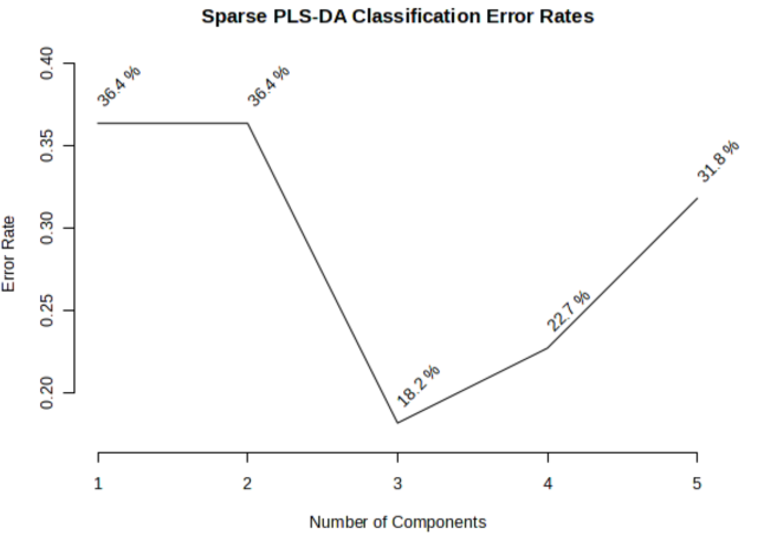


**B**

**Figure S2. Sparse Partial Least Squares-Discriminant Analysis (sPLS-DA) classification error rates evaluated using 5-fold cross-validation. (A)** sPLS-DA model performance for plasma metabolomics data. The classification error rate decreases from 64.3% with one component to 32.1% with five components, demonstrating the model's optimal performance with five components. **(B)** sPLS-DA model performance for tumor metabolomics data. The classification error rate remains constant at 36.4% for the first two components, decreases significantly to 18.2% with three components, and then increases to 22.7% and 31.8% with four and five components, respectively.

**
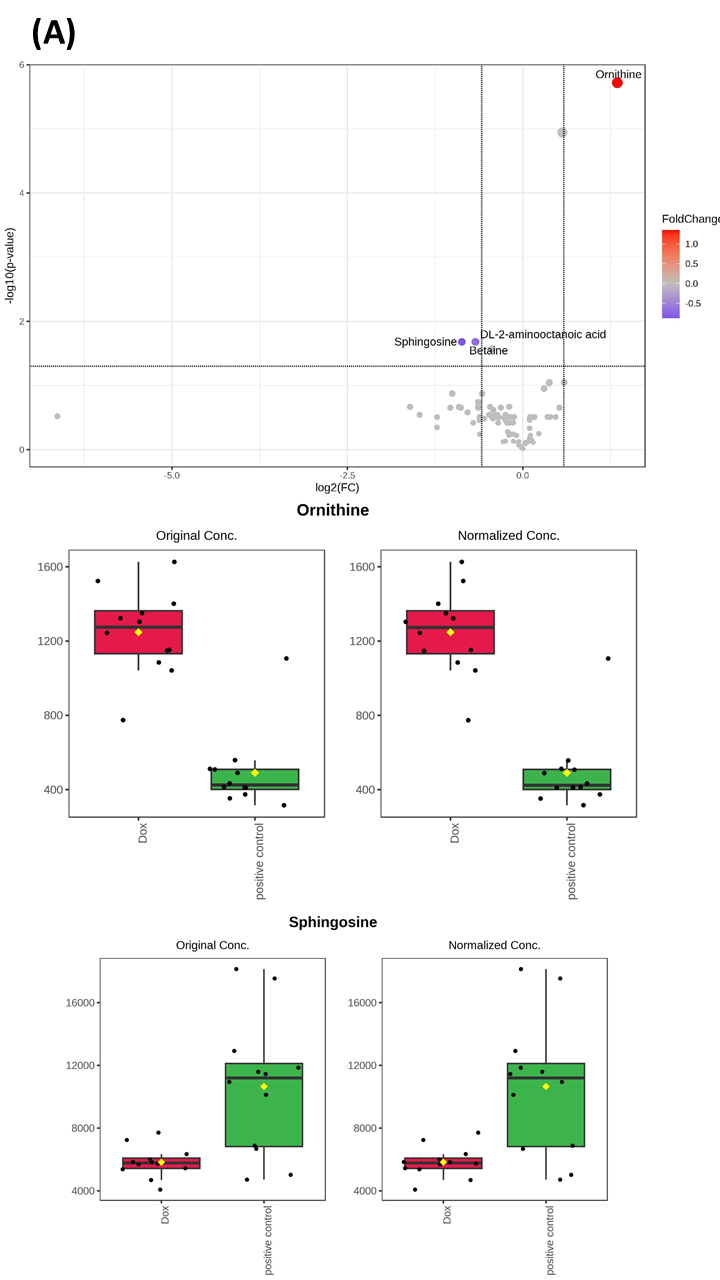
**

**
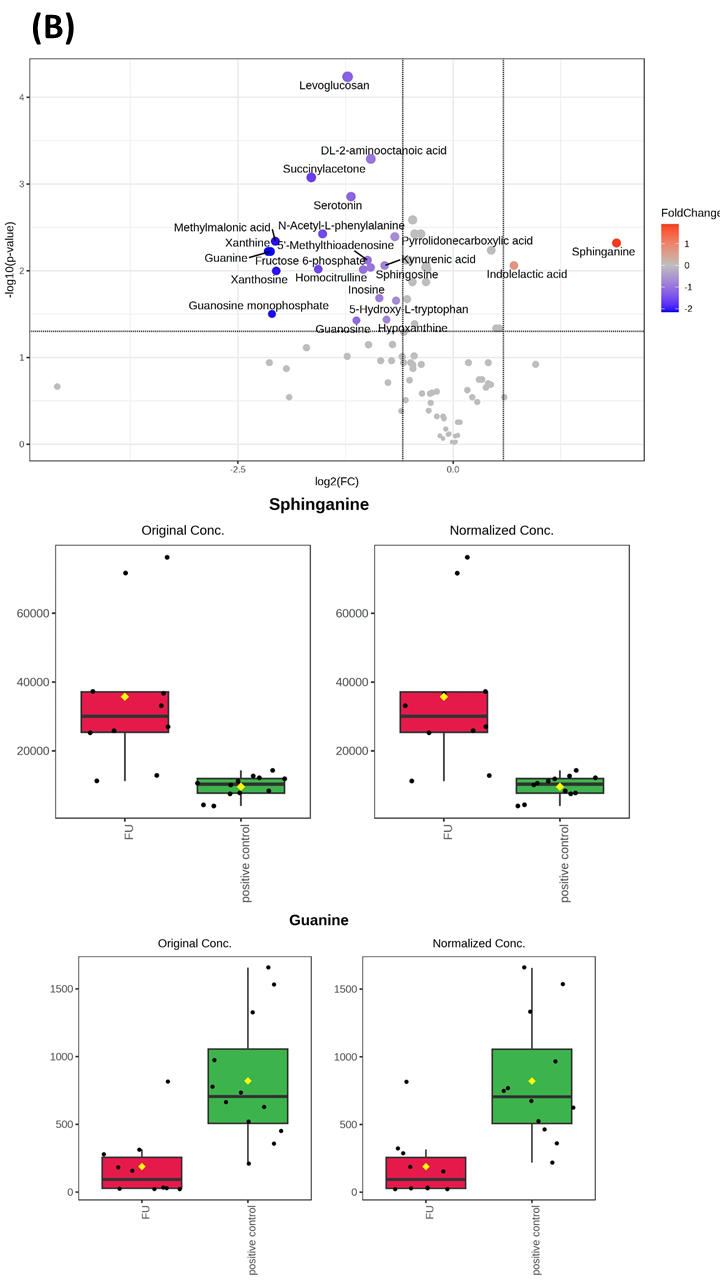
**

**
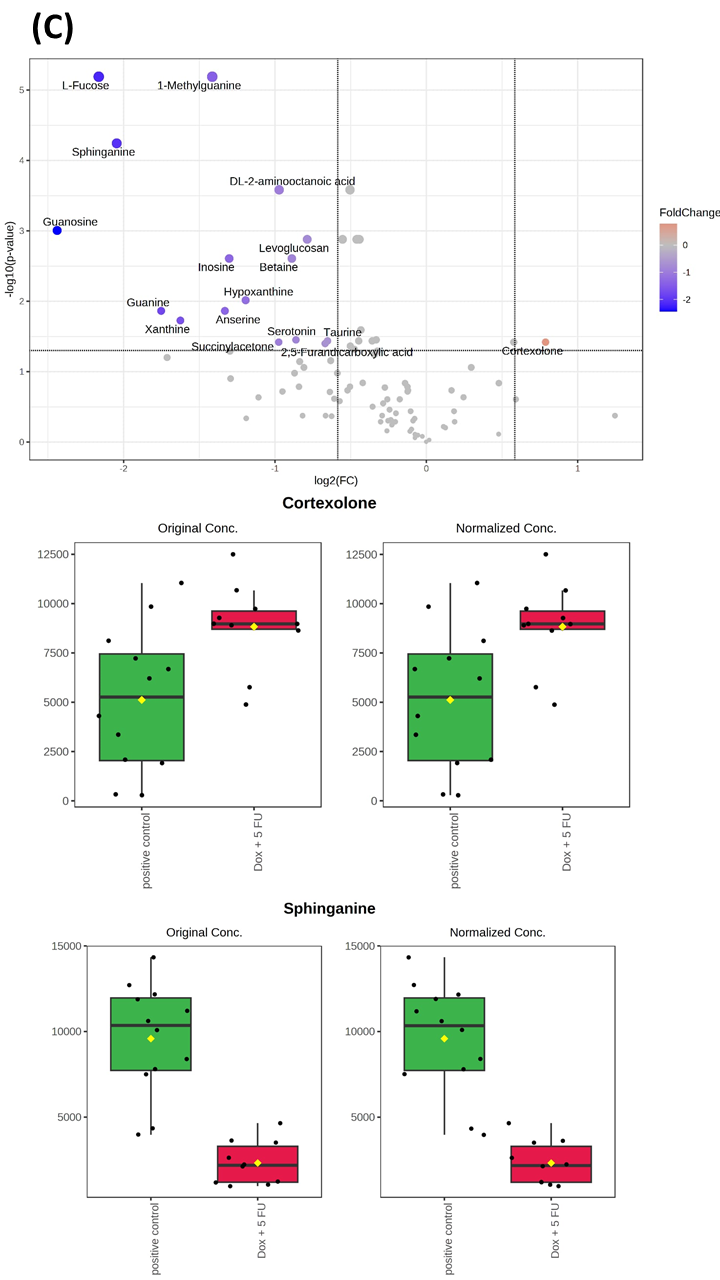
**

**
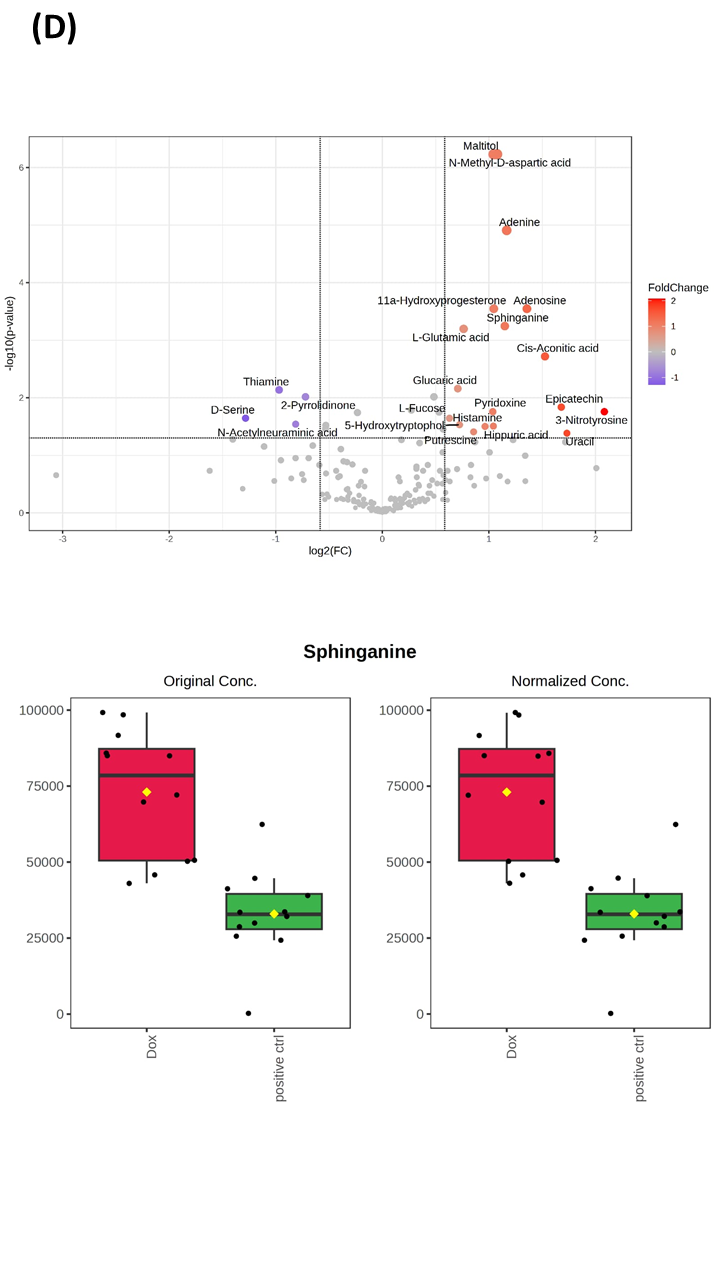
**


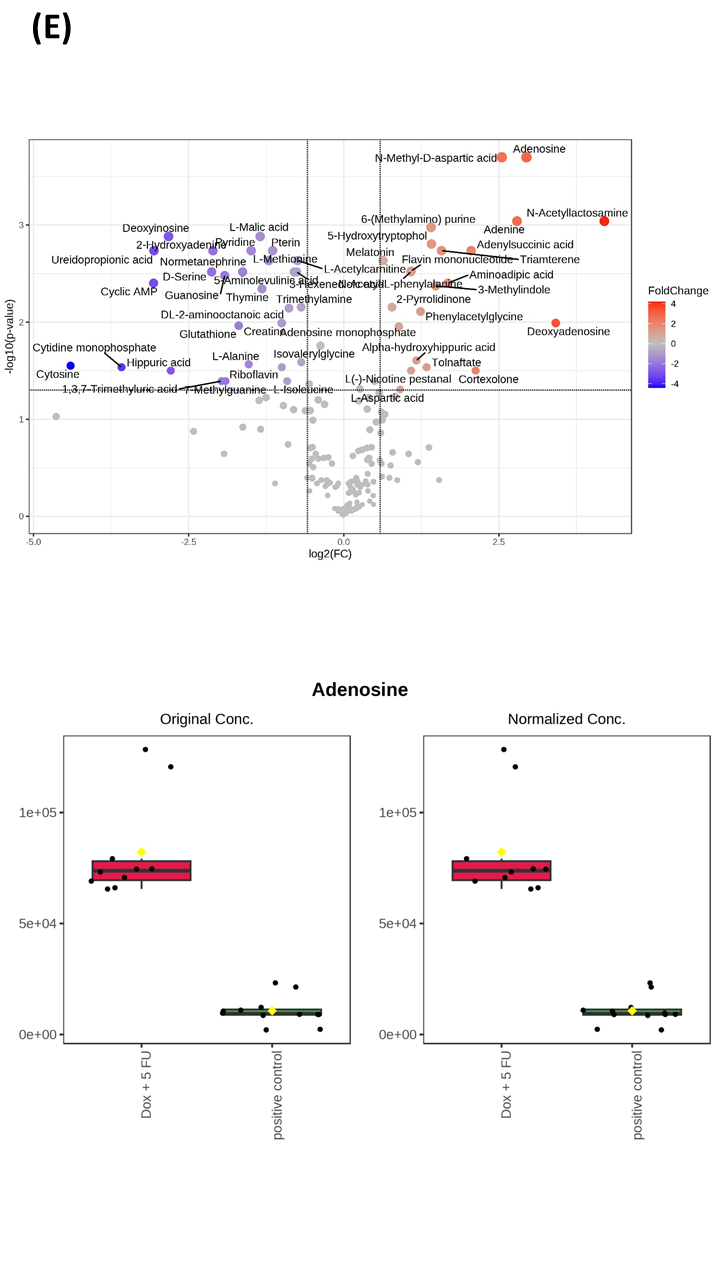


**Figure S3**. Volcano plot showing significantly altered metabolites in plasma and tumor of the different treatment groups compared to the positive control group and Boxplots for selected significant metabolites of each treatment group compared to positive control group (A) Plasma of DOX group. (B) Plasma of 5 FU group. (C) Plasma of DOX+5FU group. (D) Tumor of DOX group. (E) Tumor of DOX+ 5 FU Metabolites log2(fold change) of (Treatment /positive control) plotted against log10(p-value). Adjusted p-value <0.05, FC threshold 1.5.

## Supplementary Tables

**Table (S1): Effect Size Analysis of Tumor Volume Changes Across Treatment Groups**

| Group | Mean_Change | SD_Change | n | Pooled_SD | Cohens_d | Absolute_Reduction | Percentage_Reduction |
| --- | --- | --- | --- | --- | --- | --- | --- |
| Group 1 - DOX | 105.6178193 | 322.4519243 | 6 | 272.4939598 | -0.844448419 | 230.1070935 | 68.54036884 |
| Group 2 – 5-FU | -138.3613527 | 241.3422875 | 5 | 225.0031513 | -2.107020558 | 474.0862655 | 141.2127154 |
| Group 3 DOX + 5FU | -85.7988086 | 294.0209866 | 6 | 255.9091766 | -1.647161415 | 421.5237213 | 125.5562829 |
| Positive Control | 335.7249128 | 211.0229201 | 6 | 211.0229201 | 0 | 0 | 0 |

DOX= Doxorubicin ; 5-FU=5 Fluorouracil

The analysis was conducted using R Studio, utilizing functions from the dplyr package to summarize and analyze the data.

Table *S2*. Significantly altered metabolites in plasma analysis of treatment groups in comparison with positive control group

|  | **Metabolite Name** | ***p*-Value** | **FDR** |
| --- | --- | --- | --- |
| **Dox** | | | |
|  | Ornithine | 0.00000002 | 0.0000019 |
|  | Saccharopine | 0.00000025 | 0.00001136 |
|  | Betaine | 0.00079137 | 0.020898 |
|  | DL-2-aminooctanoic acid | 0.0010572 | 0.020898 |
|  | Sphingosine | 0.0011358 | 0.020898 |
|  | Chlorpheniramine | 0.0017627 | 0.027027 |
| **5-FU** | | | |
|  | Levoglucosan | 0.00000062 | 0.0000581 |
|  | DL-2-aminooctanoic acid | 0.000013 | 0.00060439 |
|  | Succinylacetone | 0.00004079 | 0.0012646 |
|  | L-Acetylcarnitine | 0.0001059 | 0.0024621 |
|  | Serotonin | 0.0001572 | 0.002924 |
|  | N-Acetyl-L-phenylalanine | 0.0002391 | 0.003455 |
|  | Indole | 0.00028617 | 0.003455 |
|  | 3-Hexenedioic acid | 0.00029721 | 0.003455 |
|  | Methylmalonic acid | 0.00039323 | 0.0037241 |
|  | Pyrrolidonecarboxylic acid | 0.00040044 | 0.0037241 |
|  | Guanine | 0.00072195 | 0.0061038 |
|  | Xanthine | 0.0012225 | 0.0089283 |
|  | 5'-Methylthioadenosine | 0.0015145 | 0.0089283 |
|  | Trimethylamine | 0.0015681 | 0.0089283 |
|  | Indoleacrylic acid | 0.0017757 | 0.0089283 |
|  | Glutathione | 0.0017776 | 0.0089283 |
|  | Sphingosine | 0.0018868 | 0.0089283 |
|  | L-Phenylalanine | 0.0019137 | 0.0089283 |
|  | Kynurenic acid | 0.001971 | 0.0089283 |
|  | Homocitrulline | 0.0020768 | 0.0089283 |
|  | Fructose 6-phosphate | 0.0021105 | 0.0089283 |
|  | Levulinic acid | 0.0021121 | 0.0089283 |
|  | L-Tryptophan | 0.0023643 | 0.0095601 |
|  | Xanthosine | 0.0030533 | 0.011832 |
|  | L-Norleucine | 0.0033249 | 0.012368 |
|  | 3-Methylindole | 0.003469 | 0.012408 |
|  | Indolelactic acid | 0.0038675 | 0.013321 |
|  | Sphinganine | 0.0044224 | 0.014689 |
|  | o-Tyrosine | 0.005669 | 0.01818 |
|  | 5-Hydroxy-L-tryptophan | 0.005875 | 0.018213 |
|  | Inosine | 0.0066246 | 0.019874 |
|  | Guanosine monophosphate | 0.0099762 | 0.028993 |
|  | Naproxen | 0.011768 | 0.032633 |
|  | Hypoxanthine | 0.01193 | 0.032633 |
|  | Asymmetric dimethylarginine | 0.012533 | 0.033301 |
|  | Guanosine | 0.014814 | 0.03827 |
|  | Ornithine | 0.015962 | 0.040121 |
|  | Cytidine | 0.016946 | 0.041473 |
| **Combination of Dox & 5-FU** | | |  |
|  | L-Fucose | 0.00000014 | 0.0000102 |
|  | 1-Methylguanine | 0.00000022 | 0.0000102 |
|  | Sphinganine | 0.00000382 | 0.00011836 |
|  | DL-2-aminooctanoic acid | 0.00001158 | 0.00026328 |
|  | Pantothenic acid | 0.00001416 | 0.00026328 |
|  | Guanosine | 0.00005211 | 0.00080766 |
|  | Levoglucosan | 0.00009078 | 0.0011461 |
|  | 3-Hexenedioic acid | 0.00010593 | 0.0011461 |
|  | L-Acetylcarnitine | 0.00011091 | 0.0011461 |
|  | Trimethylamine | 0.00012326 | 0.0011463 |
|  | Inosine | 0.00026584 | 0.0022476 |
|  | Betaine | 0.00037214 | 0.0028841 |
|  | Hypoxanthine | 0.0011677 | 0.0083539 |
|  | Guanine | 0.0017282 | 0.01148 |
|  | Anserine | 0.002295 | 0.014229 |
|  | Xanthine | 0.0032003 | 0.018601 |
|  | L-Norleucine | 0.0037095 | 0.020293 |
|  | L-Methionine | 0.0066636 | 0.032845 |
|  | Taurine | 0.0067103 | 0.032845 |
|  | o-Tyrosine | 0.0072293 | 0.033616 |
|  | Cortexolone | 0.0081523 | 0.036066 |
|  | Glutathione | 0.0085317 | 0.036066 |
|  | 2,5-Furandicarboxylic acid | 0.0092363 | 0.037347 |
|  | Asymmetric dimethylarginine | 0.011848 | 0.045911 |

Table S3.Significantly altered metabolites in tumor analysis of treatment groups in comparison with positive control group

|  | **Metabolite Name** | **p-Value** | **FDR** |
| --- | --- | --- | --- |
| **Dox** | | | |
|  | Maltitol | 0.00000002 | 0.00000199 |
|  | N-Methyl-D-aspartic acid | 0.00000002 | 0.00000199 |
|  | Adenine | 0.00000040 | 0.00002706 |
|  | Phenol | 0.00000170 | 0.00008709 |
|  | Adenosine | 0.00000862 | 0.00035327 |
|  | Sphinganine | 0.00002601 | 0.00069185 |
|  | 11a-Hydroxyprogesterone | 0.00002698 | 0.00069185 |
|  | L-Glutamic acid | 0.00002700 | 0.00069185 |
|  | Digoxin | 0.00008848 | 0.00201540 |
|  | Cis-Aconitic acid | 0.00011370 | 0.00233080 |
|  | L-Carnitine | 0.00056341 | 0.01007300 |
|  | 2-Pyrrolidinone | 0.00060759 | 0.01007300 |
|  | Thiamine | 0.00068007 | 0.01007300 |
|  | Glucaric acid | 0.00068792 | 0.01007300 |
|  | PC(18:1(9Z)/18:1(9Z)) | 0.00119170 | 0.01628700 |
|  | Epicatechin | 0.00134910 | 0.01728500 |
|  | Pyridoxine | 0.00165360 | 0.01994000 |
|  | 17-Hydroxyprogesterone | 0.00195920 | 0.02231300 |
|  | Isovalerylglycine | 0.00228730 | 0.02467900 |
|  | 3-Nitrotyrosine | 0.00287020 | 0.02920700 |
|  | D-Serine | 0.00309770 | 0.02920700 |
|  | N-Acetylneuraminic acid | 0.00316340 | 0.02920700 |
|  | L-Fucose | 0.00327690 | 0.02920700 |
|  | Benzaldehyde | 0.00408590 | 0.03250200 |
|  | Histamine | 0.00410830 | 0.03250200 |
|  | Hippuric acid | 0.00412220 | 0.03250200 |
|  | 5-Hydroxytryptophol | 0.00485400 | 0.03682600 |
|  | 5-Methoxytryptophol | 0.00502990 | 0.03682600 |
|  | Putrescine | 0.00580910 | 0.04106500 |
| **Combination of Dox & 5-FU** | | | |
|  | N-Methyl-D-aspartic acid | 0.00000180 | 0.00016658 |
|  | Adenosine | 0.00000198 | 0.00016658 |
|  | Atenolol | 0.00000244 | 0.00016658 |
|  | N-Acetyllactosamine | 0.00001398 | 0.00071621 |
|  | Adenine | 0.00001801 | 0.00073855 |
|  | 6-(Methylamino) purine | 0.00002612 | 0.00089250 |
|  | Deoxyinosine | 0.00004409 | 0.00116250 |
|  | L-Malic acid | 0.00004537 | 0.00116250 |
|  | 5-Hydroxytryptophol | 0.00006198 | 0.00141170 |
|  | Triamterene | 0.00008429 | 0.00172790 |
|  | Ureidopropionic acid | 0.00010608 | 0.00172800 |
|  | 2-Hydroxyadenine | 0.00011221 | 0.00172800 |
|  | Adenylsuccinic acid | 0.00011332 | 0.00172800 |
|  | Pyridine | 0.00011801 | 0.00172800 |
|  | Pterin | 0.00012706 | 0.00173640 |
|  | L-Methionine | 0.00018566 | 0.00222420 |
|  | Melatonin | 0.00019312 | 0.00222420 |
|  | L-Acetylcarnitine | 0.00019529 | 0.00222420 |
|  | Flavin mononucleotide | 0.00026428 | 0.00285150 |
|  | N-Acetyl-L-phenylalanine | 0.00030044 | 0.00295320 |
|  | D-Serine | 0.00030930 | 0.00295320 |
|  | Normetanephrine | 0.00032322 | 0.00295320 |
|  | 5-Aminolevulinic acid | 0.00033827 | 0.00295320 |
|  | 3-Hexenedioic acid | 0.00034574 | 0.00295320 |
|  | Guanosine | 0.00039311 | 0.00322350 |
|  | Cyclic AMP | 0.00049825 | 0.00386700 |
|  | Aminoadipic acid | 0.00050931 | 0.00386700 |
|  | 3-Methylindole | 0.00056567 | 0.00414150 |
|  | Thymine | 0.00062824 | 0.00444100 |
|  | Trimethylamine | 0.00101020 | 0.00688300 |
|  | 2-Pyrrolidinone | 0.00104080 | 0.00688300 |
|  | DL-2-aminooctanoic acid | 0.00110290 | 0.00706550 |
|  | Phenylacetylglycine | 0.00123170 | 0.00765170 |
|  | Creatine | 0.00171690 | 0.01006200 |
|  | Deoxyadenosine | 0.00171790 | 0.01006200 |
|  | Glutathione | 0.00188340 | 0.01072500 |
|  | Adenosine monophosphate | 0.00197970 | 0.01096800 |
|  | Benzaldehyde | 0.00320100 | 0.01726900 |
|  | Alpha-hydroxyhippuric acid | 0.00466990 | 0.02454700 |
|  | Isovalerylglycine | 0.00499040 | 0.02557600 |
|  | L-Alanine | 0.00538990 | 0.02695000 |
|  | Cytosine | 0.00570560 | 0.02784900 |
|  | Riboflavin | 0.00605350 | 0.02886000 |
|  | Tolnaftate | 0.00627200 | 0.02890000 |
|  | Cytidine monophosphate | 0.00634380 | 0.02890000 |
|  | Cortexolone | 0.00722340 | 0.03136100 |
|  | L(-)-Nicotine pestanal | 0.00725680 | 0.03136100 |
|  | Hippuric acid | 0.00734300 | 0.03136100 |
|  | 7-Methylguanine | 0.00977350 | 0.04029200 |
|  | 1,3,7-Trimethyluric acid | 0.00989420 | 0.04029200 |
|  | L-Isoleucine | 0.01019800 | 0.04029200 |
|  | Uridine | 0.01022000 | 0.04029200 |
|  | Beta-Alanine | 0.01120900 | 0.04335600 |
|  | L-Proline | 0.01277400 | 0.04849300 |
|  | L-Aspartic acid | 0.01319200 | 0.04916900 |

**
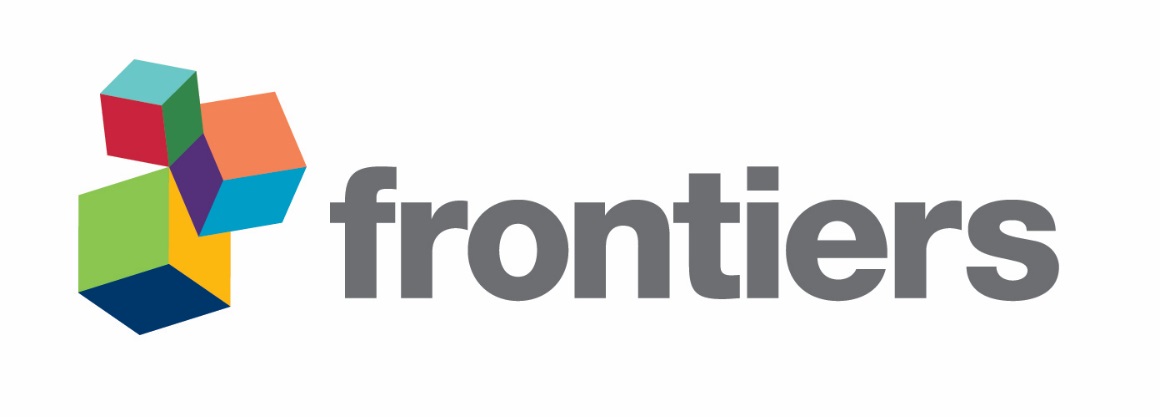
**
